# A Rare Case of Single Coronary Artery: Getting to the Heart of the Matter

**DOI:** 10.5334/jbsr.3122

**Published:** 2023-07-07

**Authors:** Ramona Mihaela Popa, Rebecca Pântea, Petrișor Macașoi, Rosana Mihaela Manea

**Affiliations:** 1 Clinical Emergency County Hospital of Brașov, RO

**Keywords:** coronary artery, coronary CTA, coronary angiography, coronary anatomy, coronary anomaly, single coronary artery, right coronary artery

## Abstract

Single coronary artery anomaly represents a particularly rare entity, which may present with variable clinical scenarios, but in most cases remains asymptomatic. It is considered to be one of the pathological states to cause sudden death, especially in young adults [1].

We hereby report a rare case of a R-III type of single coronary artery as classified by Lipton et al., which is only about 15% of all the cases of coronary anomalies. Coronary CT angiography as well as invasive coronarography provide accurate details regarding the origin, course and termination of coronary anomalies, as well as evaluation of associated coronary lesions, further guiding the optimal treatment strategy in each case.

**Teaching Point:** The main teaching point of this case report is to clearly underline the importance of coronary CT angiography in obtaining a comprehensive evaluation of coronary artery anatomy and associated coronary lesions, representing important aspects, which further guide accurate treatment and management.

## Case History

A 60-year-old female was admitted to the Emergency Department with fatigability, palpitations and low blood pressure. She was known with hypertension grade 3 and a medical history of myocardial infarction treated with balloon angioplasty of the left circumflex artery (LCX) a month ago.

**Figure 1:**
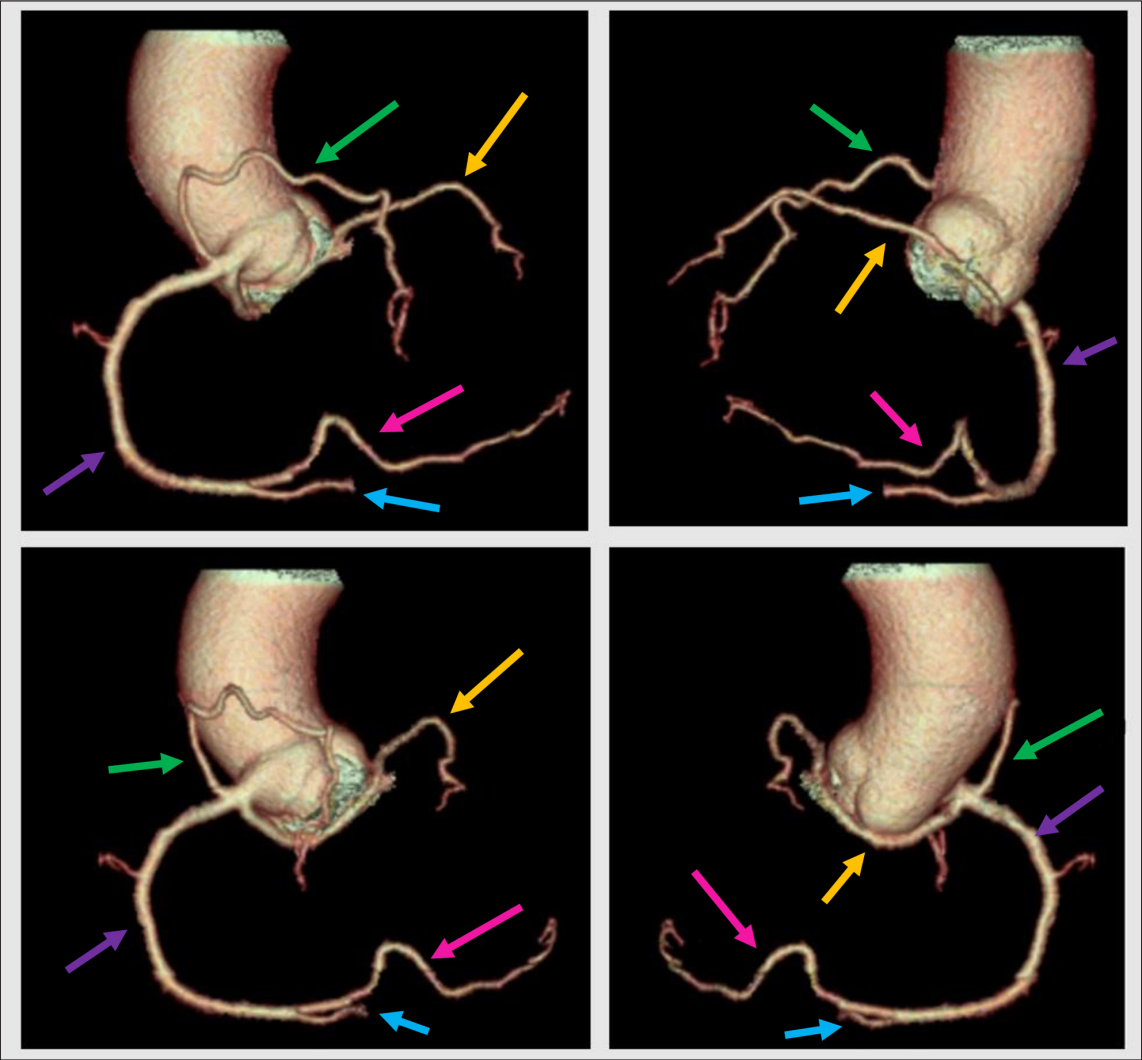


Cardiac computed tomography (CT) was performed and 3D volume rendering images illustrated a rare coronary anomaly type RIII according to Lipton’s Classification of Single Coronary Artery, characterized by a single coronary artery arising from the right sinus of Valsava, representing the right coronary artery (RCA; Figure 1, purple arrow). The posterolateral branch (PLB; Figure 1, pink arrow) presented a long vascular path, with a normal calibre. The posterior descending artery (PDA; Figure 1, blue arrow) presented a normal calibre and a normal vascular path coursing in the posterior interventricular groove.

Moreover, from the proximal segment of the RCA arose (at the same level):

- the LCX (yellow arrow), which coursed on the posterior contour of the aortic root to the left atrioventricular groove and
- the left anterior descending artery (LAD; Figure 1, green arrow), which coursed on the anterior surface of the pulmonary trunk, reaching the anterior interventricular sulcus; the LAD presented a small calibre distally as it descended to the notch of cardiac apex.

Calcium score was 0. However, due to non-calcified atherosclerotic plaques, the following stenosis were noted:

– 30–40% stenosis involving the mid segment of the RCA and
– 60–70% stenosis at the ostium of the LCX.

Therefore, the patient was referred to the catheterization laboratory for coronary angiography through the access of the right femoral artery. The cannulation of the RCA revealed a 40% stenosis of its second segment and a 80% remaining stenosis of the LCX at the ostium. Subsequently, percutaneous coronary intervention was performed painstakingly using a drug-eluting stent (2.25–18mm), which was delivered to the ostial lesion of the LCX, followed by NC balloon post-dilatation (3.0–12mm). The immediate postinterventional coronarography showed a good angiographic result, with a 25% remaining stenosis, but with adequate distal flow –TIMI 3.

Patient remained asymptomatic at 1 month and 6 months follow-up.

## Comments

Congenital anomalies involving the origin of the coronary arteries are rare entities, with variable clinical scenarios, representing a significant cause of sudden cardiac death [1].

Some anomalies, such as intramyocardial course, subendocardial course, as well as pre-pulmonic, retro-aortic, transseptal anomalies, are almost always benign based on the course alone [1]. However, if the aberrant coronary artery courses between the outflow tracts, in the case of type R/LIIB or RIII, patients are prone to major clinical complications, related to the dilatation of the great outflow tracts (aorta, pulmonary trunk) during exercise, with secondary compression of the aberrant coronary artery along their course [1].
